# Increased Release of Mercury from Dental Amalgam Fillings due to Maternal Exposure to Electromagnetic Fields as a Possible Mechanism for the High Rates of Autism in the Offspring: Introducing a Hypothesis

**Published:** 2016-03-01

**Authors:** Gh. Mortazavi, M. Haghani, N. Rastegarian, S. Zarei, S.M.J. Mortazavi

**Affiliations:** 1 Dentist, Pahlavankoshi Health Center, Ahram Health Network, Busher University of Medical Sciences, Bushehr, Iran; 2 Ionizing and Non-ionizing Radiation Protection Research Center (INIRPRC), Shiraz University of Medical Sciences, Shiraz, Iran; 3 Speech Pathology Student, Student Research Committee, School of Rehabilitation, Shiraz University of Medical Sciences, Shiraz, Iran; 4 Professor of Medical Physics, Medical Physics Department, School of Medicine, Shiraz University of Medical Sciences, Shiraz, Iran

**Keywords:** Autism spectrum disorders (ASD), Maternal Exposure, Electromagnetic fields, Mothers, Mercury release, Dental amalgam

## Abstract

According to the World Health Organization (WHO), factors such as growing electricity demand, ever-advancing technologies and changes in social behaviour have led to steadily increasing exposure to man-made electromagnetic fields.  Dental amalgam fillings are among the major sources of exposure to elemental mercury vapour in the general population. Although it was previously believed that low levels are mercury (i.g. release of mercury from dental amalgam) is not hazardous, now numerous data indicate that even very low doses of mercury cause toxicity. There are some evidence indicating that perinatal exposure to mercury is significantly associated with an increased risk of developmental disorders such as autism spectrum disorders (ASD) and attention-deficit hyperactivity disorder (ADHD). Furthermore, mercury can decrease the levels of neurotransmitters dopamine, serotonin, noreprenephrine, and acetylcholine in the brain and cause neurological problems. On the other hand, a strong positive correlation between maternal and cord blood mercury levels is found in some studies. We have previously shown that exposure to MRI or microwave radiation emitted by common mobile phones can lead to increased release of mercury from dental amalgam fillings. Moreover, when we investigated the effects of MRI machines with stronger magnetic fields, our previous findings were confirmed. As a strong association between exposure to electromagnetic fields and mercury level has been found in our previous studies, our findings can lead us to this conclusion that maternal exposure to electromagnetic fields in mothers with dental amalgam fillings may cause elevated levels of mercury and trigger the increase in autism rates. Further studies are needed to have a better understanding of the possible role of the increased mercury level after exposure to electromagnetic fields and the rate of autism spectrum disorders in the offspring.

## Introduction

### Autism as a Great Global Concern

The rapidly increasing rate of autism has caused a great concern worldwide. With a 30% increase from 1.1% in 2012, in 2014 about 1.5% of children in the United States have been diagnosed with autism spectrum disorders. This developmental disorder that affects social abilities, communication skills and cognition generally appears in the first three years of life in all races, ethnicities, and social groups. As current findings seem to be complex and inconclusive, research activities regarding the etiology of autism have increased exponentially over the last two decades. It has recently been reported that perinatal exposure to mercury is significantly associated with an increased risk of developmental disorders such as autism spectrum disorders (ASD) and attention-deficit hyperactivity disorder (ADHD). Mercury can decrease the levels of neurotransmitters dopamine, serotonin, noreprenephrine, and acetylcholine in the brain and cause neurological problems.

### Dental Amalgam in Restorative Dentistry

It is known that dental amalgam restorations are among the major sources of exposure to elemental mercury vapour in the general population. In contrast with previously accepted concepts, now numerous data indicate that even very low doses of mercury cause toxicity. Eyeson et al have previously reported that they were unable to find any correlation between perceived amalgam toxicity and mercury levels in blood and urine[1]. They stated that their study could not support this concept that exposure to mercury from amalgam may cause the development of some chronic disorders[1]. However, Kristin et al. have recently reported that based on recent epidemiological studies, the safety of mercury released from dental amalgam fillings is questionable[2]. They also reported that today, efforts are initiated to phase down or remove the use of mercury-containing amalgam in restorative dentistry.

On the other hand, a strong positive correlation between maternal and cord blood mercury levels is found in some studies. According to the report published by Palcovicova et al. in 2008, the mercury level in the cord blood was significantly associated with the number of maternal amalgam fillings and also with the time passed since the last filling. Based on these findings, they suggested that in women of reproductive age dental amalgam fillings should be used with caution to prevent increased prenatal mercury exposure[3]. Based on the finding of another study that was conducted on Wistar strain albino rats to investigate the vital tissue response in contact to dental amalgam in the mothers and their offspring, the researchers suggested that pregnant women, whenever it is possible, should delay any dental amalgam restorations to prevent possible toxic effect of mercury in the foetus[4].

### Global Increase in Exposures to Electromagnetic Fields

According to the World Health Organization (WHO), factors such as growing electricity demand, ever-advancing technologies and changes in social behaviour have led to steadily increasing exposure to man-made electromagnetic fields[5]. The rapidly increasing growth of the human exposure to electromagnetic fields (EMF) has led to growing concern about its possible health effects. Electromagnetic fields (EMF) which are produced by the motion of electrons can be generated by all electrical or electronic devices such as wireless technologies (e.g. Wi-Fi, mobile phones and cordless phones), laptop computers, microwave ovens and power lines. Over the past several years, our lab at the Ionizing and Non-ionizing Radiation Protection Research Center (INIRPRC) has performed extensive experiments on the health effects of exposure of animal models and humans to different sources of electromagnetic fields such as cellular phones[6-13], mobile base stations[14], mobile phone jammers[15], laptop computers[16], radars[7], dentistry cavitrons[17] and MRI[18, 19].

### EMF Increases the Mercury Release from Amalgam

The properties of amalgam fillings such as their ease of preparaWe have previously shown that exposure to MRI or microwave radiation emitted by common mobile phones can lead to increased release of mercury from dental amalgam fillings[18]. Furthermore, when we investigated the effects of MRI machines with stronger magnetic fields, our previous findings were confirmed entirely[20]. Our results are in line with the findings of Kursun et al. who recently found a significant increase in mercury in the teeth samples irradiated with X-ray compared to those of the control[21]. Moreover, our findings are generally in line with the results obtained in studies performed on the effect of MRI on microleakage of amalgam restorations[22, 23]. Shahidi et al. have shown that MRI is not a completely safe technique in patients with amalgam restorations[23]. A more recent study conducted by Yilmaz and Misirlioglu also showed significant differences in microleakage between the groups exposed to MRI and controls[22]. Mortazavi and Mortazavi have recently shown that a few published papers which reported no increased release of mercury after MRI, may have some methodological errors[24]. They have also reported that increased mercury release after exposure to electromagnetic fields may be risky for the hypersensitive proportion of the population and pregnant women[25-27].

### Defining and Introducing the Main Hypothesis

**Figure 1:**
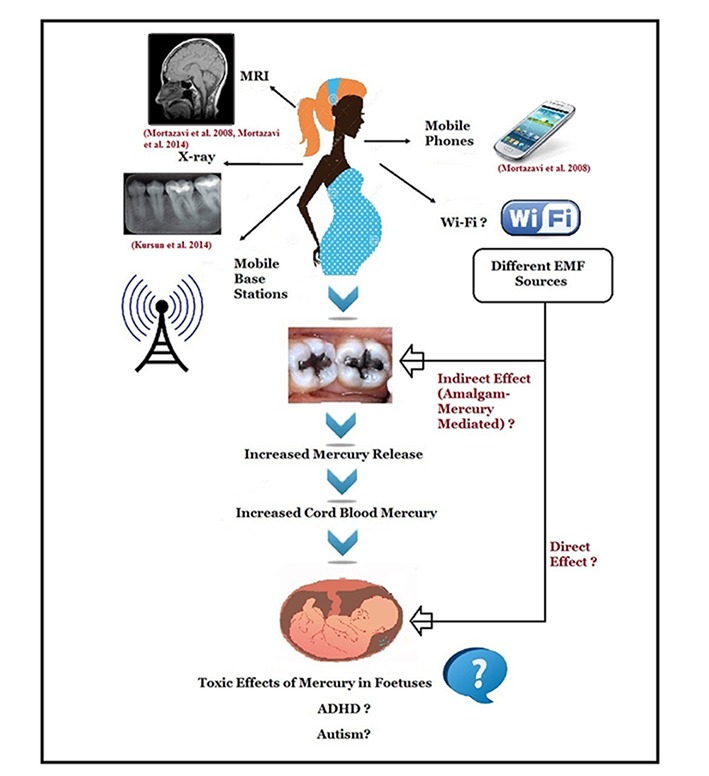
Considering the strong association between exposure to electromagnetic fields and increased mercury release from dental amalgam fillings, it can be hypothesized that maternal exposure to electromagnetic fields in mothers with dental amalgam fillings may cause elevated levels of mercury and possibly trigger the increase in autism rates.

It has been reported that the number of people diagnosed with autism spectrum disorders has been dramatically increased. In 2014, with a 30% increase from 1.1% in 2012, about 1.5% of children in the United States have been diagnosed with autism spectrum disorders. Although better diagnosis has possibly contributed to the increased rate of pervasive developmental disorders (PDDs) such as autism, it is widely believed that a real increase cannot be excluded[28]. As a strong association between exposure to electromagnetic fields and mercury level has been found in our previous studies, our findings can lead us to this conclusion that maternal exposure to electromagnetic fields in mothers with dental amalgam fillings may cause elevated levels of mercury and possibly trigger the increase in autism rates (Figure 1). Further studies are needed to have a better understanding of the possible role of the increased mercury level after exposure to electromagnetic fields and the rate of autism spectrum disorders in the offspring.
